# *Luffa cylindrica* Intercropping with *Semen cassiae*—A Production Practice of Improving Land Use in Soil Contaminated with Arsenic

**DOI:** 10.3390/plants11233398

**Published:** 2022-12-06

**Authors:** Weizhen Chen, Yanan Yang, Dele Meng, Jidong Ying, Huiyin Huang, Huashou Li

**Affiliations:** 1 College of Natural Resources and Environment, South China Agricultural University Guangzhou, Guangzhou 510642, China; 2 Guangdong Engineering Research Center for Modern Eco-Agriculture and Circular Agriculture, Guangzhou 510642, China

**Keywords:** intercropping, *L. cylindrica*, *S. cassiae*, Arsenic, safe production

## Abstract

In recent years, research on the safe utilization and green remediation of contaminated soil by intercropping has become common. In this study, the growth of an intercropping system of *Luffa cylindrica*–*Semen cassiae* in soil contaminated with medium amounts of arsenic (As) was studied using field (91.60 mg kg^−1^) and pot (83.34 mg kg^−1^) experiments. The field experiments showed that intercropping significantly increased the yield per plant of *L. cylindrica* by 27.36%, while the yield per plant of *S. cassiae* decreased by 21.66%; however, this difference was not significant. Intercropping reduced the concentration of As in all organs of *L. cylindrica* but increased the concentration of As in all parts of *S. cassiae*. The accumulation of As per plant of *L. cylindrica* was reduced by 20.72%, while that in a single plant of *S. cassiae* was increased by 201.93%. In addition, the concentration of As in the fruit of these two crops in these two planting modes was low enough to meet the National Food Safety Standard of China (GB2762-2017). In addition, the land equivalent ratio and As metal removal equivalent ratio of the intercropping mode was 1.03 and 2.34, indicating that the intercropping mode had advantages in land use and As removal. In the pot experiment, the biomass and As concentration of *L. cylindrica* and *S. cassiae* were roughly consistent with those in the field experiment. During the sampling period, intercropping reduced the concentration of As in the rhizosphere soil solution of *L. cylindrica* by 3.1–23.77%, while it increased the concentration of As in the rhizosphere soil solution of *S. cassiae* by 13.30–59.40%. The changes in pH and redox potential were also closely related to the content of water-soluble As in the rhizosphere environment, which affects the absorption of As by plants. In general, the *L. cylindrica*–*S. cassiae* intercropping system is a planting mode that can effectively treat soil that is moderately contaminated with As and remove it from the soil to an extent.

## 1. Introduction

In recent years, the use of many herbicides, preservatives, and smelting and mining operations have led to arsenic (As) pollution in a large amount of soil in China [1,2]. According to the results of the 2014 national soil pollution survey bulletin, 2.7% of China’s land is contaminated with As [3]. Owing to the advantages of low cost, easy operation, and environmental friendliness, plant extraction technology has been utilized in several field soil remediation projects [4]. At present, plants such as *Pteris vittata* [5], *Pteris cretica* [6] and *Adiantum capillus*-*Veneris* [7] have been confirmed to be As hyperaccumulators, and some As hyperaccumulators have been widely used in the field of phytoremediation of As pollution [8,9]. However, there are few economic benefits from planting hyperaccumulating plants on soil contaminated with As, for some paddy fields or cultivated lands that contaminated with As, and local farmers can hardly accept this farming method. Therefore, the goal of this study was to find a planting mode for land contaminated with As that is practical and readily acceptable by farmers.

Intercropping is a common planting method that can fully utilize water, heat, light, and other natural resources [10,11,12]. Some suitable intercropping systems can improve the land use efficiency and promote the absorption of nutrients by plants, such as the *Pisum sativum*-*Hordeum vulgare* intercropping system [13], *Solanum tuberosum*-*Phaseolus lunatus* intercropping system [14], and corn–groundnut intercropping system [15]. In terms of soil heavy metal remediation, choosing suitable plants for intercropping can ensure the production of safe edible crops on land contaminated with As and improve the phytoremediation of this soil. For example, Huang et al. [16] found that the As in rice (*Oryza sativa* L.) grains can be reduced to below the National Food Safety Standard of China (GB2762-2017) when the rice was intercropped with aquatic vegetables. Wan et al. [8] showed that the intercropping of white mulberry (*Morus alba* L.) with Chinese brake (*Pteris vittata* L.) resulted in white mulberry leaves that can meet the national feed standards. Ma et al. [9] also found that the intercropping of *P. vittata* and maize (*Zea mays* L.) is an ideal planting mode for remediating As in the soil. In addition, intercropping *Eleocharis acutangula* with *Typha domingensis* can remove barium from soil [17], and *Paspalum plicatulum*–*Axonopus affinis* [18] and *Lolium perenne*–vine plants [19] are also intercropping systems that can repair copper-contaminated soil. However, owing to the existence of As phytoremediation plants, these common intercropping models often produce limited economic benefits, which is difficult for meeting the needs of most farmers. Therefore, it is necessary to find an intercropping mode that can produce safe products without increasing environmental risks [20].

Luffa (*Luffa cylindrica*) is a common cash crop and practical vegetable in southern China. It is the primary economic source of some farmers in the region, who rely on farming for their livelihood. Wang et al. [21] found that intercropping *L. cylindrica* with *Sedum plumbizincicola* affected the absorption of cadmium by Luffa. However, few people have studied the absorption of As by *L. cylindrica* when it is planted on land contaminated with As. *S. cassiae* is a common medicinal crop in southern China. Currently, research on *S. cassiae* primarily focuses on the determination and analysis of aflatoxins [22], anthraquinone glycosides [23], or other substances in its fruit. There has been very little research on whether the content of heavy metals in the fruit of *S. cassiae* exceeds the standard.

**Figure 1:**
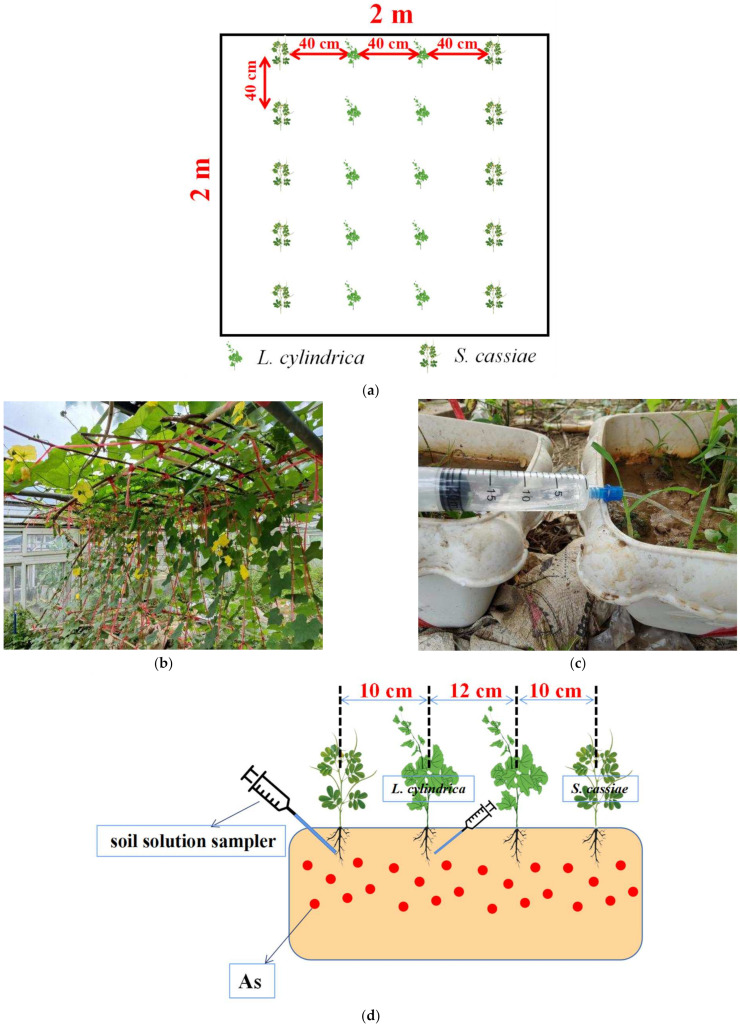
A photograph of the plots in the field experiment (**a**) and the diagram of the pot experiment and sampling of the rhizosphere soil solution (**b**–**d**).

At present, the research on whether *L. cylindrica* is planted on As-contaminated land and achieves safe production is relatively rare. *S. cassiae* belongs to leguminous plant, and when it is intercropped with *L. cylindrica*, it may provide more N elements for *L. cylindrica* [24], thus promoting the growth of *L. cylindrica*. In addition, *S. cassiae* has a high As enrichment capacity. When it is intercropped with *L. cylindrica*, it may reduce the As absorption of *L. cylindrica*. On the other hand, even if the As concentration of *S. cassiae* fruit exceeds the edible standard, it can also be used to produce pillows or other products, thus producing certain economic benefits. Some photos of the research are shown in Figure 1. Thus, in our study, *L. cylindrica* and *S. cassiae* were selected for planting experiments on land contaminated with As to identify an intercropping model that can achieve safe production on As-contaminated land and remove As from the soil to a certain extent. We hypothesized the following: (1) intercropping with *S. cassiae* would reduce the content of As in *L. cylindrica*; (2) intercropping with *L. cylindrica* would promote the absorption of As by *S. cassiae* to an extent; and (3) this intercropping system would primarily affect the absorption of As by *L. cylindrica* and *S. cassiae* by changing the concentration of water soluble As in the rhizosphere environment of plants.

## 2. Results

### 2.1. Field Experiment

#### 2.1.1. Biomass and Yield of Plants

For *L. cylindrica*, compared with monoculture system, the intercropping mode significantly increased the biomass of *L. cylindrica* stems and leaves by 88.10% and 32.64%, respectively, (*p* < 0.05) compared with the monoculture system (Table 1). The yield of *L. cylindrica* per plant in the intercropping mode was 27.36% higher than that in the monocropping mode (*p* < 0.05).

Intercropping significantly reduced the stem biomass of *S. cassiae* by 31.50% (*p* < 0.05), while the biomass of other parts of *S. cassiae* in the two planting systems had no significant differences. Compared with the monoculture mode, the per ha yield of *L. cylindrica* and *S. cassiae* in the intercropping mode significantly decreased (*p* < 0.05), which was caused by the significant decrease in the number of plants planted in the intercropping mode.

In addition, the LER of this intercropping mode was 1.03. This also proved that the intercropping of *L. cylindrica* and *S. cassiae* has advantages in yield and land use.

#### 2.1.2. Levels of As in Different Parts of the Plants and the BCA

As shown in Table 2, compared with the monoculture, intercropping significantly reduced the concentrations of As in the roots, leaves, and fruit of *L. cylindrica* by 22.78%, 31.38%, and 50.0%, respectively (*p* < 0.05). In the intercropping system, the BCA per plant and per ha of *L. cylindrica* significantly decreased compared with the monocropping system by 20.72% and 60.42%, respectively (*p* < 0.05).

Intercropping significantly increased the concentrations of As in the roots, leaves, pods, and fruit of *S. cassiae* compared with those in the monoculture system, which increased by 166.50%, 344.44%, 645.45% and 416.67%, respectively (*p* < 0.05). The BCA per plant of *S. cassiae* in the intercropping system was 201.93% higher than that in the monoculture system (*p* < 0.05), and there was no significant difference between the BCA per ha of *S. cassiae* in the two planting systems.

In addition, the value of the MRER of the intercropping system reached 2.34. This also proved that the intercropping of *L. cylindrica* and *S. cassiae* has apparent advantages in the removal of heavy metals.

### 2.2. Pot Experiment

#### 2.2.1. Biomass and Yield of *L. cylindrica* and *S. cassiae*

**Figure 2:**
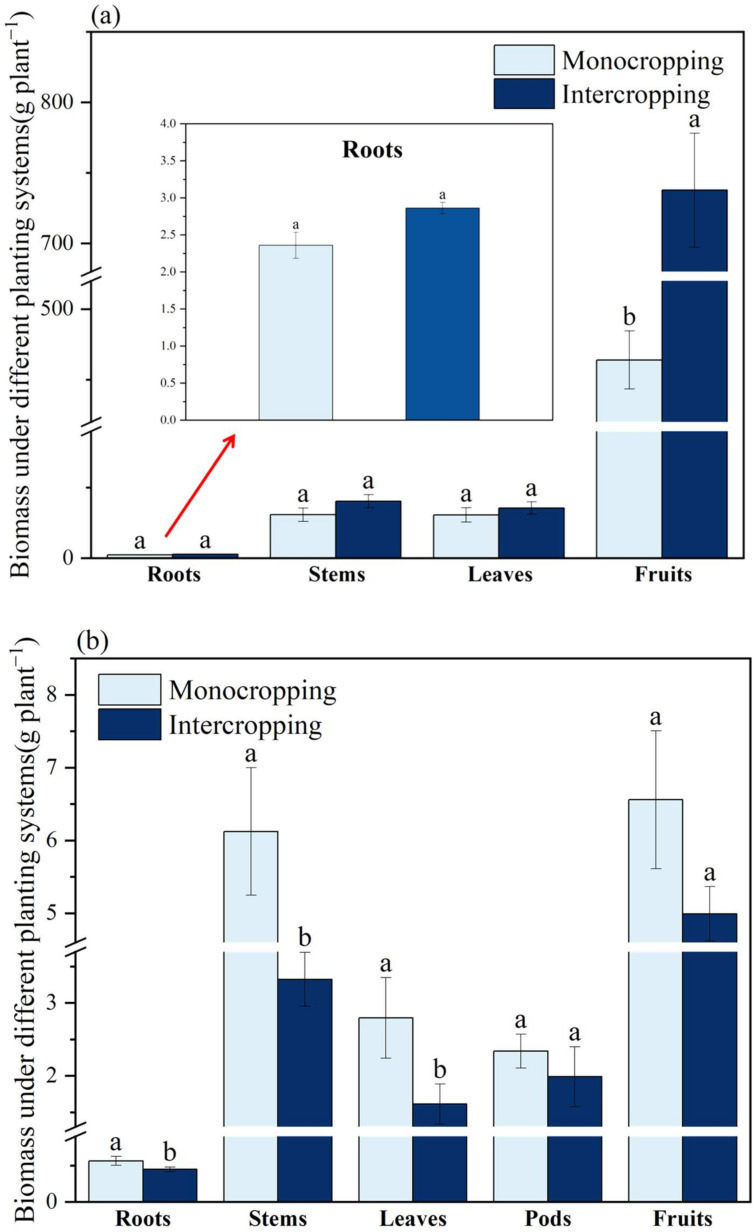
Biomass of *L. cylindrica* (**a**) and *S. cassiae* (**b**) in different planting systems. Different lowercase letters indicate significant (*p* < 0.05) differences between the treatments according to Duncan’s test.

The biomass of all parts of *L. cylindrica* increased in the intercropping system compared with the monoculture system, but only the increase in the fruit biomass was significantly different, which increased by 59.04% compared with the monoculture mode (*p* < 0.05) (Figure 2a).

The intercropping mode of *S. cassiae* significantly reduced the biomass of roots, stems, and leaves by 20.70%, 45.73%, and 42.19%, respectively (*p* < 0.05), compared with the monoculture mode (Figure 2b). The difference in the biomass of the *S. cassiae* pods and fruit between the monoculture and intercropping systems were not significantly different.

#### 2.2.2. Concentration of As in Different Parts of *L. cylindrica* and *S. cassiae* and the BCA

**Figure 3:**
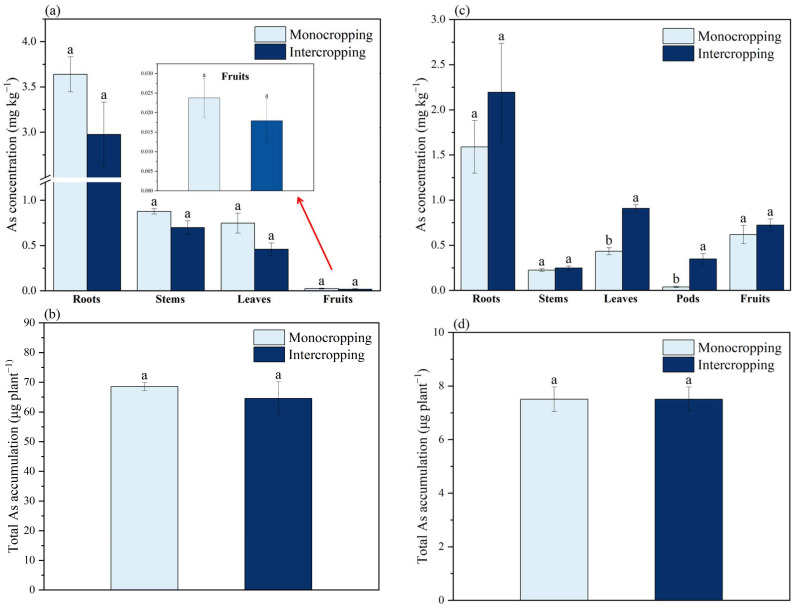
As concentrations and total Cd accumulation in *L. cylindrica* (**a**,**b**) and *S. cassiae* (**c**,**d**) under different planting systems. Different lowercase letters indicate significant (*p* < 0.05) differences between the treatments according to Duncan’s test.

Compared with the monoculture system, the intercropping system increased the As concentration in various parts of *L. cylindrica*, but this increase did not reach a significant difference level (Figure 3a). Compared with *S. cassiae* in the monoculture system, intercropping significantly increased the contents of As in its leaves and pods by 109.47% and 815.79%, respectively (*p* < 0.05) (Figure 3c). The difference in the BCA of *L. cylindrica* and *S. cassiae* in these two planting modes did not reach a significant level (Figure 3b,d).

#### 2.2.3. Concentration of As and the pH and Eh of the Rhizosphere Soil Solution

**Figure 4:**
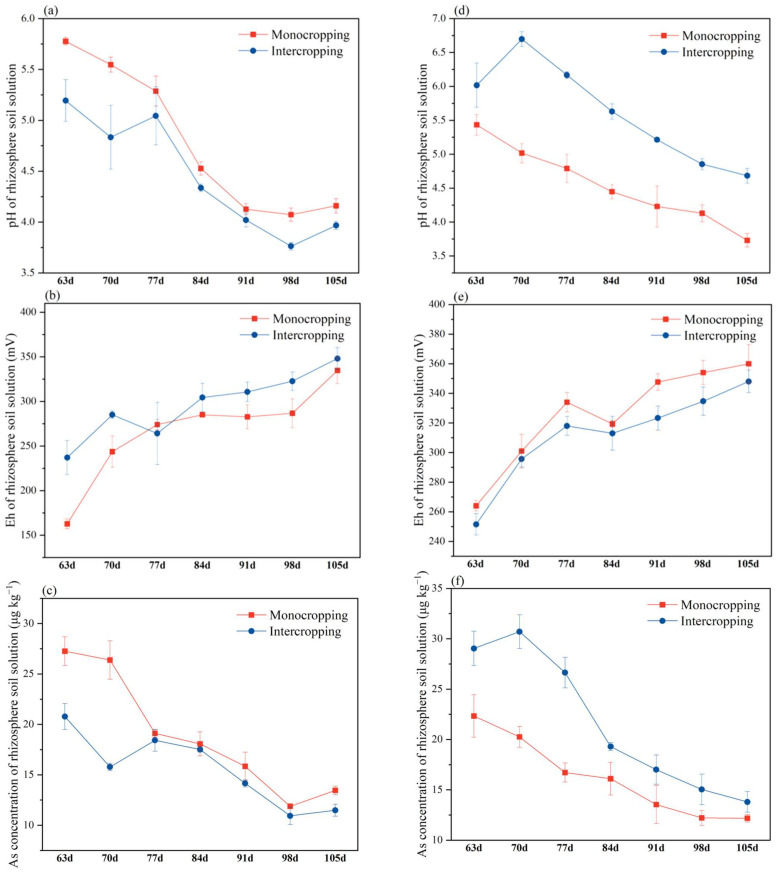
Changes in the pH, Eh, and As concentration in the rhizosphere soil solution of *L. cylindrica* (**a**–**c**) and *S. cassiae* (**d**–**f**) during the sampling period. Different lowercase letters indicate significant (*p* < 0.05) differences between the treatments according to Duncan’s test.

For *L. cylindrica*, the pH of the rhizosphere soil solution in the monoculture system decreased from 5.78 to 4.16, while that in the intercropping system decreased from 5.19 to 3.97 during the entire sampling period (Figure 4a). The Eh of the rhizosphere soil solution of *L. cylindrica* in the monoculture system during the entire sampling period showed an upward trend and increased from 162.67 mV to 334.67 mV, whereas that in the intercropping system increased from 237.01 mV to 348.00 mV (Figure 4b). The concentration of As in the rhizosphere soil solution of *L. cylindrica* in the monoculture system decreased from 27.26 μg kg^−1^ to 13.45 μg kg^−1^, while that in the intercropping system decreased from 20.78 μg kg^−1^ to 11.48 μg kg^−1^ (Figure 4c). During the entire sampling period, the intercropping mode decreased the content of As in the rhizosphere soil solution for *L. cylindrica* compared with the monocropping system, with a range of decrease of 3.1–23.77%.

The pH of the rhizosphere soil solution in the monoculture system of *S. cassiae* decreased from 5.43 to 3.73, while that in the intercropping system decreased from 6.02 to 4.65 (Figure 4d). The Eh of *S. cassiae* in the rhizosphere soil solution in the monoculture system increased from 264.0 mV to 360.0 mV, whereas that of the rhizosphere soil solution of *S. cassiae* in the intercropping system increased from 251.5 mV to 348.0 mV (Figure 4e). The concentration of As in the rhizosphere soil solution of *S. cassiae* in the monoculture system decreased from 22.33 μg kg^−1^ to 12.18 μg kg^−1^, while that in the intercropping system decreased from 29.04 μg kg^−1^ to 13.80 μg kg^−1^ (Figure 4f). During the entire sampling period, the intercropping mode decreased the As content of the rhizosphere soil solution for *S. cassiae* compared with the monocropping system, with a range of decrease of 13.30–59.40%.

## 3. Materials and Methods

### 3.1. Soil Characterization

The field experiment was located in Xinjiang Town, Shaoguan City, Guangdong Province, China (24°29′31″ N; 113°48′39″ E), which is close to the Dabaoshan Mine. For a long time, wastewater from Dabaoshan Mine has been used for irrigation in this area, resulting in soil contaminated with As in this area. According to the World Reference Base for Soil Resources [25], the soil type in this area is Anthrosol. The relevant information about the soil used for the field experiments is as follows: pH 5.54, 91.60 mg kg^−1^ of total As, 2.81 g kg^−1^ of total N, 0.75 g kg^−1^ of total P, 20.31 g kg^−1^ of total K, 15.86 g kg^−1^ of total C, and 17.67 g kg^−1^ of organic matter. According to the standards from the National Soil Environmental Quality Risk Control (GB15618-2018), the farmland soil in this area is moderately polluted with As.

We collected soil contaminated with As using hoes and soil samplers (with scales) and used a soil layer of 0–20 cm from contaminated farmland near the field experiment for the pot experiments (24°29′35″ N; 113°46′26″ E). The soil in this area had also been irrigated by wastewater from the Dabaoshan mine for a long time. We used rectangular pots for the pot experiments (48 cm long × 24.2 cm wide × 17.5 cm high). The soil samples collected in the field were naturally dried and were then screened to ensure that the soil particle size was approximately 2 mm. They were then placed inside the pots (6.5 kg per pot). The basic physical and chemical properties of the soil for pot experiments were as follows: pH 5.45, 83.34 mg kg^−1^ of total As, 2.27 g kg^−1^ of total N, 0.92 g kg^−1^ of total P, 21.56 g kg^−1^ of total K, 16.52 mg kg^−1^ of total C, and 21.99 g kg^−1^ of organic matter.

### 3.2. The L. cylindrica and S. cassiae Seedlings

In both experiments, the seeds of *L. cylindrica* were provided by the Guangdong Academy of Agricultural Sciences (Guangzhou, China), and the seeds of *S. cassiae* were of conventional varieties.

### 3.3. Experimental Design

In the field experiment, the experimental area was divided into 2 × 2 m plots with an interval of 1.5 m between the plots to avoid the interaction between different treatments. The plot division and planting selection were completely random, and each treatment was repeated three times. The treatments included *L. cylindrica* monoculture, *S. cassiae* monoculture, and *L. cylindrica* intercropped with *S. cassiae*. Each plot in the monoculture treatments contained four rows of plants with a total of 20 plants, and the spacing between rows and between plants were 40 cm. In the intercropping treatments, two lines of *L. cylindrica* and two lines of *S. cassiae* were planted in each plot, and the spacing between rows and between plants were 40 cm. The specific planting method is shown in Figure 1a. The fields were managed based on local agronomic practices, and the irrigation water met the general standard of agricultural water. The field experiment began on 28 June 2022, and the two crops were harvested on 30 September 2022.

The pot experiments were conducted in the greenhouse of an ecological farm at South China Agricultural University (Guangzhou, China). There were three treatments in the pot experiments: (1) *L. cylindrica* monoculture. Two Luffa plants were planted in each pot, and the plants were spaced 25 cm apart; (2) *S. cassiae* monoculture. Two *S. cassiae* plants were planted in each pot, and the plants were spaced 25 cm apart; and (3) intercropping of *L. cylindrica* and *S. cassiae*. Two *L. cylindrica* plants and two *S. cassiae* plants were planted in each pot. The specific planting method is shown in Figure 1d. The pot experiment began on 12 April 2021, and the two crops were harvested on 28 July 2021. When the growth of the two plants approached the mature stage, their rhizosphere soil solution was extracted weekly using a soil solution sampler (MacroRhizon flflex; 10 cm porous material, 0.2 μm pore size, and 4.5 mm outer diameter; Rhizosphere Research Products B.V., Wageningen, The Netherlands). The specific method of extraction is shown in Figure 1d.

### 3.4. Sampling and Analysis

#### 3.4.1. Sampling and Analysis

After samples of these two plants in the field and pot experiments were harvested, *L. cylindrica* was divided into four parts, including the roots, stems, leaves, and fruit. For the collection of plant roots, the complete roots and soil around them should be taken out as much as possible when sampling, and then the roots should be carefully separated from the soil around them by using tools such as a fine brush. Place the separated roots in water for careful cleaning to remove the residual soil on the root surface. During the cleaning process, try to keep the roots floating, and pick out all roots after washing to avoid the loss of roots. After washing and drying with tap water, the fresh weight of each part was weighed. The plant sample was then placed in an electric blast dryer (HK-750AS+, Guangzhou Jinzong Machinery Equipment Co., Ltd., Guangzhou, China) and was dried at 105 °C for 30 min; it was further dried to achieve a constant weight at 70 °C. *S. cassiae* was divided into five parts, including the roots, stems, leaves, pods, and fruit, and the samples were treated in the same manner as those of *L. cylindrica*. In this study, the biomass of the fruit of both plants was expressed in fresh weight, and the biomass of the other parts was expressed in dry weight. The biomass and yield of plants per ha was then calculated.

#### 3.4.2. Determination of As

##### Total As Concentration in *L. cylindrica* and *S. cassiae*

The dried plant samples were ground into powder using a stainless steel mill and were then digested by microwave digestion (MARS6, CEM Corporation, Matthews, NC, USA) using a mixed solution of nitric acid (HNO_3_) and hydrogen peroxide (H_2_O_2_) [26,27,28]. The control sample (CDHK-GBW (E) 100349) and the blank sample with rice flour as the primary component were used for quality control. The measuring standard as specified by the Chinese government (GB 5009.11-2014) was used to determine the total As in food, and the concentration of As in the sample was determined by atomic fluorescence spectrometry (ZAF-3100). The rate of recovery of As in the reference materials was >85%.

##### Determination of the As Concentration, pH, and Oxidation-Reduction Potential (Eh) in the Rhizosphere Soil Solution

After the rhizosphere soil solution was removed, its pH and Eh values were measured using a pH meter (SX-620, Shanghai San-Xin Instrumentation, Inc., Shanghai, China) and Eh meter (SX-630, Shanghai San-Xin Instrumentation, Inc., Shanghai, China), respectively. The soil solution was then passed through a 2 mm filter membrane, and the pH was adjusted to <2 with 5% HCl. Atomic fluorescence spectrometry (ZAF-3100) was used to measure the As concentration in the soil solution. This method was that same as that used for the plants.

### 3.5. Data Analysis

#### 3.5.1. Bioconcentration Amount (BCA)

The BCA value can be used to evaluate the ability of plants to absorb heavy metals in contaminated soil [26,27] In our study, the BCA value was used to express the total concentration of As accumulated by plants in μg or g.

(1) $$\mathrm{BCA}=\sum B_{p}{*C}_{p}$$

where C_P_ (mg kg^−1^) and B_P_ (g) in this formula represent the As content and relevant biomass of each organ of *L. cylindrica* (root, stem, leaf, and fruit) and *S. cassiae* (root, stem, leaf, pod, and fruit).

#### 3.5.2. Land Equivalent Ratio (LER)

The LER is an index that can be used to evaluate whether the intercropping system has a particle yield advantage [29]. The specific calculation formula of LER is as follows:

(2) $$\mathrm{LER}=\left(\frac{Y_{\mathrm{iL}}}{Y_{\mathrm{mL}}}\right)+\left(\frac{Y_{\mathrm{iS}}}{Y_{\mathrm{mS}}}\right)$$

The $Y_{\mathrm{iL}}$ and $Y_{\mathrm{iS}}$ in this formula refer to the yields of *L. cylindrica* and *S. cassiae* in the intercropping mode, respectively, and $Y_{\mathrm{mL}}$ and $Y_{\mathrm{mS}}$ refer the yields of *L. cylindrica* and *S. cassiae* in the monocrop mode, respectively, in kg. If the LER > 1, it means that this intercropping mode has a good land use effect. Furthermore, the intercropping mode has little value in land use when its LER less than 1 [26,29,30].

#### 3.5.3. Metal Removal Equivalent Ratio (MRER)

The MRER is an index that can be used to evaluate whether the intercropping system can remove heavy metals from contaminated soil. In this study, the MRER was used to evaluate whether this intercropping system can remove As in soil contaminated with the element [26,30]. It is calculated by the following formula:

(3) $$\mathrm{MRER}=\left(\mathrm{BCA}\_\mathrm{iL}/\mathrm{BCA}\_\mathrm{mL}\right)+\left(\mathrm{BCA}\_\mathrm{iS}/\mathrm{BCA}\_\mathrm{mS}\right)$$

${\mathrm{BCA}}_{\mathrm{iL}}$ and ${\mathrm{BCA}}_{\mathrm{iS}}$ in this fomula refer to the BCA values of *L. cylindrica* and *S. cassiae* in the intercropping mode, respectively, and ${\mathrm{BCA}}_{\mathrm{mL}}$ (mg) and ${\mathrm{BCA}}_{\mathrm{mS}}$ (mg) refer to the BCA values of *L. cylindrica* and *S. cassiae* in the monoculture mode, respectively.

All analyses of data were conducted using SPSS 20.0 software (IBM, Inc., Armonk, NY, USA). Differences in the means among treatments were analyzed using the least significant difference method at *p* < 0.05. All figures were generated using Origin 2022b software (OriginLab, Northampton, MA, USA).

## 4. Discussions

### 4.1. Growth Status of L. cylindrica and S. cassiae

The results of the field and pot experiments showed that compared with the monoculture system, the biomass of each part of *L. cylindrica* in the intercropping system increased, particularly in yield. This could be because *S. cassiae* is a legume and can promote the transfer of nitrogen to non-leguminous plants [24]. Thus, *L. cylindrica* in the intercropping system had a richer source of nutrients. Similar conclusions have been drawn in the research by Tang et al. [31]. However, the biomass of *S. cassiae* showed the opposite trend, and the biomass from intercropping was smaller than that of the monoculture. This could be because *L. cylindrica* has a larger biomass and resource competitive advantage, which is similar to the research of Kang et al. [32]. In the pot experiment, the root system of *L. cylindrica* is much larger than that of *S. cassiae*, so *L. cylindrica* has a greater root competitive advantage and has a wider growth space when intercropping with *S. cassiae*, which also leads to the increase of *L. cylindrica* biomass and the significant decrease of *S. cassiae* biomass in the intercropping system. It should be noted that although the yield of *S. cassiae* in the intercropping mode decreased compared with that of the monocropping mode, the difference was not significant. Therefore, the LER of this intercropping system is slightly greater than 1.0, which is a planting mode that can efficiently utilize land contaminated with As to a certain extent, which is similar to the conclusions drawn by Wan et al. [8] and Kang et al. [26].

### 4.2. Levels of As in L. cylindrica and S. cassiae

Our results show that the *L. cylindrica*–*S. cassiae* intercropping system can significantly reduce the concentration of As in all parts of *L. cylindrica* as intercropping with *S. cassiae* restricts the transfer of As from the belowground to the aboveground of *L. cylindrica*, which can also reduce the risk of As exposure to an extent; a similar phenomenon has been noted in a study by Mensah et al. [33]. In addition, the concentration of As in the *L. cylindrica* fruit in these two planting systems (monocropping: 0.08 mg kg^−1^; intercropping: 0.04 mg kg^−1^) met the National Food Safety Standard of China (GB2762-2017) (0.5 mg kg^−1^). This is because melon crops generally have a relatively weak ability to absorb heavy metals. Ma et al. [9] also found that the grain of maize under different *Pteris vittata L.*–maize intercropping conditions satisfied foods standard for As concentration. Although the biomass of *S. cassiae* had decreased in the intercropping system, the concentration of As in each part of *S. cassiae* had increased. This indicated that *S. cassiae* is better able to become enriched with As than *L. cylindrica*. In addition, the As concentration of *S. cassiae* fruits in the two planting systems (monocropping: 0.24 mg kg^−1^; intercropping: 1.24 mg kg^−1^) also met the National Food Safety Standard of China (GB2762-2017) (2 mg kg^−1^). After referring to the As content threshold of polished rice (0.2 mg kg^−1^) stipulated by the FAO [34], we found that in this intercropping system, the fruits of *L. cylindrica* can be safely eaten; however, the As concentration of *S. cassiae* fruits exceeds this particular threshold. Therefore, for the application of this type of intercropping in the world, we advise that the fruit of *L. cylindrica* is safely usable for food, and the fruit of *S. cassiae* should be used as raw materials for making pillows or other products, so as to ensure that this intercropping system can produce certain economic benefits while removing As in the soil, and has better popularization.

It is worth noting that the MRER of this intercropping system reached 2.34, which has a very clear advantage for removing heavy metals. Therefore, the fruits of the two plants produced by this intercropping system have economic benefits, and this intercropping model can remove As from contaminated land to some extent. This is economically beneficial and has popular appeal; it is superior to some intercropping systems, such as rice–aquatic vegetables [16], cassava (*Manihot esculenta* L. Crantz)–peanut (*Arachis hypogea* L.) (MRER = 1.25) [30], or rice–*Sesbania* (MRER = 1.11) [32].

### 4.3. Changes in the pH, Eh, and As Content in the Rhizosphere Soil Solution of L. cylindrica and S. cassiae

By extracting the rhizosphere soil solution and measuring the content of As in the soil solution, the influence of intercropping on the availability of As in the soil can be explored to further explain the potential risk of As in the soil environment [35,36]. In this study, the effects of intercropping on the accumulation of As in plants were explored by observing the changes in the pH, Eh, and As concentration in the rhizosphere soil solution [35]. The concentration of As in the rhizosphere soil solution of *L. cylindrica* under intercropping was lower than that under monocropping, which indicated that intercropping reduces the bioavailability of As in the rhizosphere environment of *L. cylindrica* [37]. Under intercropping conditions, compared with monocropping, the pH and Eh in the rhizosphere soil solution of *L. cylindrica* showed a downward and upward trend, respectively, indicating that the pH and Eh were closely related to the content of As in the rhizosphere soil solution [38]. With the decrease in pH, the content of water-soluble As also decreased [39]. On the other hand, the decrease in the rhizosphere pH may also increase the activity of some toxic substances, such as aluminum [40] or cadmium [41] and may also affect the effectiveness of some micronutrients in the rhizosphere [42]. However, because the *L. cylindrica* of this variety has a large biomass and the decline of pH in this study is not significant, this change will not have a significant negative impact on the growth of *L. cylindrica*.

The trend of As concentration, pH, and Eh in the rhizosphere soil solution of *S. cassiae* in different planting systems was opposite to that shown in the *L. cylindrica* rhizosphere soil solution because the increase in pH value and decrease in Eh value affected the mobilization of As in the soil [43] and promoted the release of As from the soil [44,45]. Therefore, the root system of *S. cassiae* absorbs more As from the soil. This result can also provide a theoretical basis for improving the phytoremediation of soil pollution [46].

## 5. Conclusions and Prospects

This study explored the feasibility of intercropping *L. cylindrica* and *S. cassiae* as an agronomic measure to achieve safe production and soil remediation to some extent in soil that is contaminated with medium concentrations of As. The results showed that the fruit of *L. cylindrica* and *S. cassiae* in the monoculture system and intercropping system could meet the National Food Safety Standard of China (GB2762-2017), but whether it can be popularized worldwide still needs further research. In general, this intercropping mode can realize the effective utilization of As-contaminated soil and produce certain economic value, and it has obvious advantages in removing As from the soil. The pot experiment showed that the intercropping system primarily regulated the absorption of As by *L. cylindrica* and *S. cassiae* by changing the pH, Eh, and water-soluble As content of the rhizosphere environment. Future research should explore the specific mechanism of As absorption by this intercropping system to *L. cylindrica* and *S. cassiae* through the determination of different forms of As in the rhizosphere environment and other relevant indicators. Such research is underway.

## Figures and Tables

**Table 1:** Biomass and yield of *L. cylindrica* and *S. cassiae* under monocropping and intercropping systems in the field experiment.

Crops	Items	Organs	Plant Mode	
			Monocropping	Intercropping
*L. cylindrica*	Biomass (g plant^−1^)	Roots	2.33 ± 0.20 a	2.98 ± 0.30 a
		Stems	19.84 ± 1.96 b	37.32 ± 4.52 a
		Leaves	30.36 ± 0.61 b	40.27 ± 3.14 a
		Fruits	1223.91 ± 61.83 b	1558.75 ± 37.44 a
	Yield (kg ha^−1^)		61,195.63 ± 3091.49 a	38,968.75 ± 936.07 b
	LER		1.03	
*S. cassiae*	Biomass (g plant^−1^)	Roots	3.57 ± 0.34 a	2.67 ± 0.11 a
		Stems	18.89 ± 0.33 a	12.94 ± 0.84 b
		Leaves	4.35 ± 0.33 a	3.76 ± 0.60 a
		Pods	13.82 ± 1.72 a	10.82 ± 1.24 a
		Fruits	34.76 ± 6.89 a	27.23 ± 3.53 a
	Yield (kg ha^−1^)		1737.92 ± 344.62 a	680.83 ± 88.37 b

Abbreviations: LER—Land equivalent ratio. All values are presented as mean ± standard error (*n* = 3). An independent sample *t*-test was used to determine whether the two mean values obtained for the monocropping and the intercropping differ significantly. Different lowercase letters indicate a significant (*p* < 0.05) difference between the treatments according to Duncan’s test. All values are presented as mean ± standard error (*n* = 3). The same below.

**Table 2:** Concentration of As in plant organs and BCA under the monocropping and intercropping systems in the field experiment.

Crops	Items	Organs	Plant	
			Monocropping	Interplanting
*L. cylindrica*	As concentration (mg kg^−1^)	Roots	5.18 ± 0.12 a	4.00 ± 0.30 b
		Stems	1.76 ± 0.36 a	0.88 ± 0.041 a
		Leaves	2.39 ± 0.09 a	1.64 ± 0.07 b
		Fruits	0.08 ± 0.01 a	0.04 ± 0.01 b
	BCA (μg plant^−1^)		210.12 ± 2.95 a	166.58 ± 5.80 b
	BCA (g ha^−1^)		10.51 ± 0.15 a	4.16 ± 0.15 b
*S. cassiae*	As concentration (mg kg^−1^)	Roots	2.06 ± 0.41 b	5.49 ± 0.78 a
		Stems	0.29 ± 0.01 a	0.28 ± 0.03 a
		Leaves	0.36 ± 0.03 b	1.60 ± 0.21 a
		Pods	0.11 ± 0.02 b	0.82 ± 0.09 a
		Fruits	0.24 ± 0.02 b	1.24 ± 0.18 a
	BCA (μg plant^−1^)		22.28 ± 2.0 b	67.27 ± 10.68 a
	BCA (g ha^−1^)		1.11 ± 0.11 a	1.68 ± 0.27 a
	MRER		2.34	

Abbreviations: BCA—Bioconcentration amount; MRER—Metal removal equivalent ratio.

## Data Availability

Not applicable.
